# Caught in the Crossfire: Unmasking the Silent Renal Threats of Tyrosine Kinase Inhibitors in Chronic Myeloid Leukemia †

**DOI:** 10.3390/cancers17010092

**Published:** 2024-12-30

**Authors:** Maria Benkhadra, Rola Ghasoub, Reem Hajeomar, Awni Alshurafa, Nabeel Mohammad Qasem, Giuseppe Saglio, Jorge Cortes, Islam Elkonaissi, Rasha Kaddoura, Mohamed A. Yassin

**Affiliations:** 1 Department of Pharmacy, National Center for Cancer Care and Research, Hamad Medical Corporation, Doha P.O. Box 3050, Qatar; 2 College of Pharmacy, Qatar University, Doha P.O. Box 2713, Qatar; 3 Department of Hematology and Bone Marrow Transplant, National Center for Cancer Care and Research, Doha P.O. Box 3050, Qatar; 4 Department of Clinical and Biological Sciences, University of Turin, 10124 Turin, Italy; 5 Division of Hematology and SCT, Georgia Cancer Center, Augusta, GA 30912, USA; 6 Shaikh Shakhbout Medical City, Abu Dhabi P.O. Box 11001, United Arab Emirates; 7 Pharmacy Department, Heart Hospital, Hamad Medical Corporation, Doha P.O. Box 3050, Qatar

**Keywords:** chronic myeloid leukemia, chronic kidney disease, tyrosine kinase inhibitors, renal impairment, nephrotoxicity, adverse drug reaction

## Abstract

Background: Renal adverse drug reactions (ADRs) associated with tyrosine kinase inhibitors (TKIs) in the treatment of chronic myeloid leukemia (CML) are relatively rare, and there is currently no standardized protocol for their management. Therefore, this study aimed to summarize renal ADRs related to TKIs use in CML and propose an evidence-based approach to monitor and manage these ADRs. Methods: A systematic literature review was performed to identify renal ADRs associated with TKIs in CML. Two authors screened the search results and extracted data from 37 eligible studies. These findings were then used to develop a scheme for clinicians to monitor and manage these ADRs. Results: Overall, imatinib seemed to be significantly linked to renal adverse events compared to other TKIs, and switching to dasatinib or nilotinib significantly improved renal function. Similar events were reported with bosutinib, although they were not statistically significant. However, most of the renal events reported on dasatinib were described as nephrotic syndrome that resolved with switching to imatinib. Few cases were reported with nilotinib that described tumor lysis syndrome (TLS)-related kidney injury. Conclusions: Recommendations include monitoring for progressive decline in the estimated glomerular filtration rate with imatinib, nephrotic syndrome with dasatinib, and TLS with nilotinib. Additionally, holding the offending TKI and managing renal ADRs according to local guidelines were adopted more frequently than reducing the TKI dose.

## Simple Summary

The survival of patients with chronic myeloid leukemia (CML) has improved with the use of tyrosine kinase inhibitors (TKIs). However, TKI-related kidney side effects present challenges, especially in older patients, who are predisposed to kidney impairment. This systematic review provides the first assessment of kidney side effects that are associated with the use of TKIs in CML. It also summarizes the practical recommendations for medication selection to minimize kidney toxicity. It revealed that, compared to other TKIs, imatinib is more strongly associated with kidney side effects over time, while dasatinib and nilotinib are generally linked to better kidney outcomes. However, dasatinib is associated with nephrotic syndrome (NS) and an increased risk of heart and lung toxicities in patients undergoing hemodialysis. Suggested approaches include testing for protein in the urine to screen for NS when administering dasatinib. For nilotinib, a gradual increase in dosage is recommended to reduce the risk of tumor lysis syndrome (TLS). Patients with existing reduced kidney function or risk factors such as high blood pressure and diabetes require additional precautions, including the use of alternative TKIs when possible and avoiding kidney-toxic medications. This review highlights the importance of consistent and detailed reporting of kidney side effects and stresses the need for additional research on long-term effects.

## 1. Introduction

The incorporation of anti-BCR-ABL tyrosine kinase inhibitors (TKIs) into the treatment landscape for chronic myeloid leukemia (CML) has positively altered patients’ long-term survival [1,2]. Imatinib, a first-generation TKI, significantly improved the prognosis and survival of patients with CML [1]. Moreover, the second, third, and fourth generations of TKIs (bosutinib, dasatinib, nilotinib, ponatinib, and asciminib) improved response rates and allowed hematologists to select the most appropriate TKI for each individual patient [1,3]. Despite the established efficacy of TKIs, practical recommendations for managing their associated toxicities remain limited [4,5].

Kidney dysfunction and CML exhibit several commonalities, particularly as both conditions frequently co-exist in the elderly population. These diseases often present with non-specific symptoms such as fatigue and anemia, which complicate early diagnosis and management. Moreover, CML can directly impact renal function through leukemic infiltration of the kidneys. The use of TKIs in CML treatment, although effective, adds another layer of complexity, as these agents are associated with an increased risk of cardiovascular disease (CVD), which can subsequently lead to or exacerbate kidney dysfunction given the shared risk factors of hypertension (HTN), diabetes mellitus (DM), and obesity [5,6,7]. The interplay between these conditions is further complicated by the challenges of polypharmacy, increasing the likelihood of drug–drug interactions and difficulties in maintaining treatment adherence. This interrelationship necessitates the need for a practical guide and a careful, patient-centered approach to the identification, prevention, and management of renal dysfunction in CML patients on TKIs.

Nephrotoxicity can manifest as either acute kidney injury (AKI) or chronic kidney disease (CKD) [8]. According to the Kidney Disease Improving Global Outcomes (KDIGO), AKI is defined as an increase in serum creatinine (SCr) by ≥50% within 7 days, an increase in SCr by ≥0.3 mg/dL (≥26.5 µmol/L) within 48 h, or oliguria with or without markers of structural damage (e.g., proteinuria). As for CKD, it is defined as abnormalities in kidney structure or function lasting more than 3 months, with implications for health [8]. The National Cancer Institute (NCI) has developed the Common Terminology Criteria for Adverse Events (CTCAE) to provide a consistent description and grading of renal adverse drug reactions (ADRs) associated with therapeutic agents (Table 1) [9].

According to the Food and Drug Administration (FDA) Adverse Events Reporting System (FAERS), a total of 2715 cumulative renal and urinary ADRs were reported with TKIs in CML patients from 2001 until 20 June 2023 [10]. The highest number of reports were with imatinib (n = 1009), followed by bosutinib (n = 173), dasatinib (n = 299), nilotinib (n = 636), ponatinib (n = 573), and asciminib (n = 25) (see Table 2) [10]. Although FAERS is largely governed by reporting trends with medications, it indicated that kidney dysfunction events with TKIs among CML patients were largely overlooked in the literature. The incidence, patterns, and severity of renal ADRs of TKIs are inconsistent. This further emphasizes the need for guidance in the management of renal ADRs caused by anti-BCR-ABL TKI. This review aims to systematically describe the TKIs-associated renal ADRs and offer evidence-based recommendations for detecting, monitoring, and managing renal adverse events.

## 2. Methodology

A comprehensive literature review that encompassed human studies published in English from their inception until June 2023 was performed and repeated on 5 April 2024. The primary sources employed were PubMed and Embase databases, excluding Medline as a source to avoid duplication. The search strategy employed the following terms: “imatinib”, “dasatinib”, “asciminib”, “ponatinib”, “nilotinib”, “bosutinib”, “renal insufficiency”, “chronic myeloid leukemia”, “chronic kidney”, and “acute kidney failure”. The detailed search strategy is available in Supplementary Materials (Text S1). All studies identified by the search strategy were transferred to EndNote to ensure that all the excluded studies were recorded and referenced. Duplicates were manually removed and validated via EndNote.

The screening process was performed by one author (MB) and subsequently validated by two other authors (AA and RH). Original human studies reporting renal ADRs among TKI-treated CML patients as well as TKI pharmacokinetics in renal impairment [only section with studies on healthy subjects] were eligible for inclusion. Animal studies, reviews, studies on the use of TKIs in non-CML malignancies, and those that did not report renal outcomes were excluded. The data extraction was performed by two authors (AA and RH) and validated by a third author (MB). Discrepancies were resolved through consensus meetings, where the authors discussed and addressed any disagreements. To ensure accuracy and consistency, a standardized data collection Excel sheet was used. The collected data included the characteristics of the studies (i.e., design and publication type), population characteristics, sample size, specific TKI used (dose and duration when available), renal outcomes (CKD [new/worsened], AKI, nephrotic syndrome, or effects of renal impairment on TKI pharmacokinetics), and management approach for renal ADRs. The included studies were compiled and categorized into four main themes:

1. Pharmacokinetic Changes of TKIs in Renal Impairment: comparative analysis of findings with published data from tertiary sources;
2. Renal ADRs Associated with Single TKIs: studies reporting renal ADRs related to one specific TKI;
3. Renal ADRs Associated with Two TKIs: studies documenting renal ADRs associated with two different TKIs;
4. Renal ADRs Associated with Three or More TKIs: studies detailing renal ADRs involving three or more TKIs.

The Joanna Briggs Institute (JBI) tool was utilized for observational studies and case reports to assess the risk of bias in the included studies [11]. Missing data were addressed through multiple strategies to ensure comprehensive and accurate results. When essential data were missing, efforts were made to contact study authors to request additional information or clarification. Studies with significant amounts of missing data that could not be resolved were excluded from the final analysis. The review protocol was registered with PROSPERO (CRD42024575376).

## 3. Results

### 3.1. Studies Selection and Characteristics

**Figure 1:**
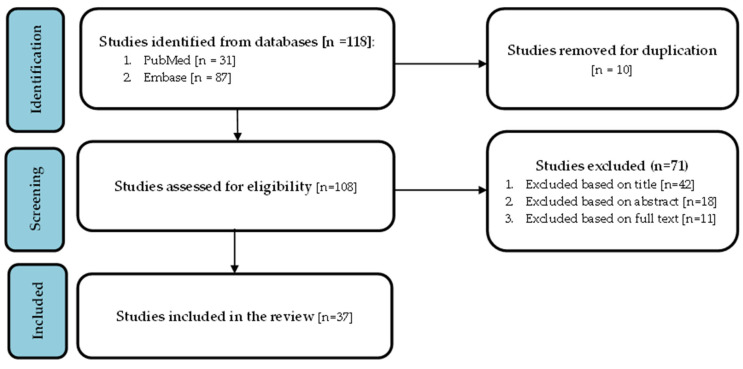
Record screening process (PRISMA flow chart).

From a total of 108 records (after duplicate removal), 37 studies met the eligibility criteria [12,13,14,15,16,17,18,19,20,21,22,23,24,25,26,27,28,29,30,31,32,33,34,35,36,37,38,39,40,41,42,43,44,45,46,47,48], and 71 studies were excluded. Figure 1 (PRISMA flow chart) demonstrates the screening process. All excluded studies are referenced in Supplementary Table S2. Table 3 summarizes the pre-existing pharmacokinetic data on TKIs in CML. The publication types from our results included 25 full-text articles, and 12 were conference abstracts (details in Table 4, Table 5, Table 6 and Table 7). The studies included were case reports and series (n = 17), observational (n = 14; 12 retrospective and 2 prospective cohort) studies, pharmacokinetic studies (n = 2), a mixture of case report and pharmacokinetic study (n = 1), analysis of FAERS reports (n = 1), a long-term analysis of multiple trials (n = 1), and a post-marketing surveillance (n = 1). Aside from the pharmacokinetic studies, most of the studies described renal ADRs with one (n = 15), two (n = 9), or three or more (n = 11) TKIs.

### 3.2. Pharmacokinetics of CML TKIs in Renal Impairment

According to the pharmacokinetics of TKIs (Table 3), namely the exposure (measured as Area Under the Curve, AUC) of a TKI, imatinib and bosutinib are the most affected by renal impairment because >10% of each is excreted by the kidney [49,50,51,52]. Therefore, both TKIs require dose adjustments in renal impairment (Table 3).

The exposures (AUC) of dasatinib, nilotinib, and ponatinib have minimal renal excretion and, therefore, exert less impact on renal function [53,54,55,56,57,58]. Thus, none of them require a renally adjusted dose (Table 3). Interestingly, although >10% of asciminib is excreted in urine and the AUC is increased by 54% in patients with creatinine clearance (CrCl) of 15–30 mL/min, the maximum serum concentration slightly increases by only 6% [59,60]. Hence, dose adjustments are not recommended in patients with CrCl > 15 mL/min [60].

Two of the included studies (Table 4) described the effects of pre-existing renal impairment on the pharmacokinetics of bosutinib and asciminib compared to patients with normal renal function [12,13]. Bosutinib exposure (AUC) significantly increased in patients with moderate (CrCl 30–50 mL/min) and severe (CrCl < 30 mL/min) renal impairment [12]; therefore, dose reduction is recommended for patients with CrCl < 50 mL/min [51]. Asciminib’s pharmacokinetics and safety profile remained clinically insignificant in severe renal impairment, though safety assurance is limited [13].

**Table 3:** Pharmacokinetic data on FDA-approved TKIs in CML as reported by FDA label and drug monographs.

TKI	Pharmacokinetics in Renal Impairment	Dose Adjustment in Renal Impairment	Renal ADRs Reported
Imatinib	A 1.5- to 2-fold increase in exposure (AUC) in patients with mild (CrCl 40–59 mL/min) and moderate (CrCl 20–39 mL/min) renal impairments [49]. Thirteen percent excreted in urine [50].	Recommended starting dose [49]: Mild renal impairment (CrCL = 40–59 mL/min): Doses greater than 600 mg are not recommended. Moderate renal impairment (CrCl 20–39 mL/min): 50% reduction in the usual initial dose and increased as tolerated. Doses greater than 400 mg are not recommended. Severe renal impairment (CrCl <20 mL/min): use with caution. Reports of tolerance of 100 mg/day in two patients and exposure was similar to 400 mg in normal kidney function.	0.1 to 1% in initial label [49]. Significant in post-marketing surveillance [49]. Renal toxicity was added as a warning in 2017 label [49].
Bosutinib	A 1.4- and 1.6-fold increase in exposure (AUC) in patients with moderate (CrCl 40–59 mL/min) and severe (CrCl 20–39 mL/min) renal impairments, respectively [51]. Thirteen percent excreted in urine [52].	Recommended starting dose [51]: Moderate renal impairment (CrCl 30–50 mL/min): 400 mg PO once daily. Severe renal impairment (CrCl <30 mL/min): 300 mg PO once daily.	Significant percentage (>20%) [51]. Renal toxicity was added as a warning in 2021 label [51].
Dasatinib	No clinically relevant effect on AUC (exposure) in CrCl 21.6 to 342.3 mL/min [54]. Less than 4% excreted in urine [53].	No dosage adjustment required [54].	0.1 to 1%. [54] Nephrotic syndrome reported in post-marketing surveillance [54].
Nilotinib	Unknown pharmacokinetics [55]. Not excreted in urine [56].	No dosage adjustment is likely required (not studied) [56].	1–5% [56].
Ponatinib	No clinically relevant effect on AUC (exposure) in CrCl > 30 mL/min [58]. Five percent excreted in urine [57].	No dose adjustment in CrCl > 30 mL/min, but not studied in renal function below that [58].	Not mentioned in labels; increased serum creatinine recorded at 20% [58].
Asciminib	No clinically relevant effect on AUC (exposure) on CrCl >30 mL/min, but increased by 54% in CrCl >13 to <30 mL/min with only 6% increase in maximum serum concentration [60]. Eleven percent excreted in urine [59].	No dose adjustment in CrCl > 15 mL/min, but not studied in renal function below that [60].	Not mentioned in labels; increased serum creatinine recorded at 31% [60].

Abbreviations: AUC: Area Under the Curve, CrCl: Creatinine Clearance.

**Table 4:** Pharmacokinetic studies in patients with renal impairment.

Study		Population and Design	Effect of Renal Impairment on TKI
Bosutinib	Abbas R, et al. (2016) *Full text*) [12]	Multicenter open-label parallel group design on patients with mild, moderate, and severe renal impairment versus healthy individuals.	For moderate renal impairment, the AUC increased by 35% (significant), and the Cmax increased by 28% (not significant). For severe renal impairment, the AUC increased by 60% (significant), and the Cmax increased by 34% (not significant). Dose adjustments may be needed in CrCl < 50 mL/min.
Asciminib	Hoch M, et al. (2021) *Full text*) [13]	Phase-I study of the safety of a single oral dose of asciminib according to renal function: Cohort 1: eGFR ≥90 mL/min. Cohort 2: severe renal impairment (eGFR < 30 mL/min). Cohort 3: moderate renal impairment (eGFR 30 to 60 mL/min). Cohort 4: mild renal impairment (eGFR 60 to < 90 mL/min).	Absorption phase and Cmax are similar between severe renal impairment and normal renal function. Plasma disposition is slower in severe renal impairment, resulting in significantly higher systemic exposure, longer half-life, and reduced clearance, although the increase in exposure was not clinically relevant. Renal impairment may not have a clinically meaningful effect on the exposure or safety profile of asciminib.

### 3.3. Renal Outcomes from Studies Reporting on One TKI

Fifteen studies reported renal outcomes with the use of a single TKI (seven case reports, six observational cohort studies, one analysis of FAERS reports, and one post-marketing surveillance study) [14,15,16,17,18,19,20,21,22,23,24,25,26,27,28]. Most of the reported renal events were documented with imatinib (n = 11 studies) when compared to dasatinib (n = 2 studies) or nilotinib (n = 2 studies). Table 5 describes the details of all the eligible studies in this section.

**Table 5:** Studies reporting renal events with one and two TKIs in CML patients.

Study		Design	Population	Comorbidities/Risk Factors	TKI	Renal Events	Interventions	Conclusion
Imatinib	Celestino JL, et al. (2023) *Conference abstract* [14]	Observational, cross-sectional	N = 160 CML patients (phase of disease was not specified)	SAH and older age	***Results were only reported for imatinib; dose was not specified***	The occurrence of renal impairment was significantly associated with imatinib use in both univariate and multivariate analyses. Additional risk factors included older age and SAH. ⇒ **New onset CKD** ⇒ **Time to event is not mentioned**	Not specified	Imatinib is significantly associated with a decline in GFR and incidence of CKD.
	Nakahara R, et al. (2021) *Full text* [21]	Case report	An 88-year-old man with CML	Older age, CHF, HTN, DM, CKD stage G5A3, history of left renal cancer and left nephrectomy	Imatinib 100 mg and increased to 200 mg after 3 weeks	Renal function deteriorated after the initial dose increased to 200 mg/day (CKD stage G5A3 to dialysis) and led to the discontinuation of imatinib. Imatinib was restarted at 200 mg/day with dialysis (stable) and TDM guidance. TDM repeated on day 28 from imatinib resumption; the steady-state plasma concentration was confirmed [trough level was 2514 ng/mL, i.e., lower than the mean trough concentration of 3180 ng/mL (associated with a higher grade 3/4 AEs)]. Renal function did not worsen with no other Aes, and efficacy was not affected at day 35. ⇒ **Worsening of CKD to dialysis** ⇒ **Time to event: around 1 week (extracted from figure)**	Discontinuation of the offending drug (imatinib). Initiation of emergency dialysis. Management of fluid overload and HTN. Careful re-administration of imatinib guided by TDM.	Imatinib use in CKD led to worsening from pre-dialysis G5A3 to dialysis. The use of TDM for imatinib during dialysis allowed safe and effective administration of imatinib in one case report. Evidence is insufficient to provide TDM guidance of therapy.
	Singh A, et al. (2023) *Full text could not be obtained* [15]	Prospective cohort study	N = 55 patients with CP-CML	HTN and DM	***Results were only reported for imatinib; dose was not specified***	Seven percent developed AKI. Sixteen percent developed new CKD. eGFR significantly reduced (from 74 to 59 mL/min/1.73 m2) and led to a significant subsequent significant reduction in Hgb levels (10.9 to 9 g/dL) 12 months post imatinib therapy. ⇒ **New onset CKD and renal anemia** ⇒ **Time to event: measured at 12 months**	Not specified	Imatinib treatment significantly reduces eGFR and Hgb levels, with a strong negative correlation between the two decreases.
Imatinib	Mestroni R, et al. (2011) *Conference abstract* [22]	Case report	An 85-year-old with CP-CML	IHD, MI, aortic stenosis (moderate), SAH, BPH, and CKD III stage	Imatinib 300 mg daily.	AKI 2 weeks post starting imatinib therapy leading to dialysis with acute heart failure and refractory fluid overload. Renal obstruction was ruled out.Patient passed away a month later despite hematological response. ⇒ **Worsening of CKD to dialysis** ⇒ **Time to event: 2 weeks**	Dialysis Other measures were not specified	Imatinib therapy led to AKI requiring dialysis, fluid overload, and subsequent cardiac decompensation. Patient passed away.
	Thiem U, et al. (2021) *Full text* [24]	Case report	A 57-year-old man with CML	SAH, DM, CAD, PAD, and stroke	Imatinib 400 mg daily.	No worsening renal outcomes or significant ADRs while on imatinib therapy and after the second kidney transplant. ⇒ **No renal event** ⇒ **Time to event: not applicable**	N/A *(2nd kidney transplant was due to deterioration prior to initiation of imatinib)*	Imatinib therapy did not cause significant deterioration in renal condition in a CKD patient who underwent his second transplantation while on imatinib. Risk factors: history of CKD with transplantation + dialysis, DM, and SAH.
	Moura M, et al. (2019) *Full text* [16]	Retrospective cohort	N = 55 patients with CP-CML.	HTN (33.4%), DM (26.7%), CV disease (11.7%), hypothyroidism (10%) and CKD (13.3%).	Imatinib (most started at 400 mg/day; dose ranged between 300–800 mg/day).	19 patients (22.6%) developed decline in renal function (GFR decline and creatinine level elevation). Of those patients, n = 18 had grade 1 toxicity, and n = 1 had grade 3 toxicity, while 77.4% of the study population had no renal side effects. ⇒ **New onset CKD, worsening of CKD, possible renal anemia** ⇒ **Time to event: measured at 2 years**	Not specified	Imatinib therapy led to decline in renal function over time in one-fifth of the study population. Ruling out other causes of anemia (non-CML/imatinib hematologic toxicity) is recommended.
Imatinib	Sakurai M, et al. (2016) *Conference abstract* [17]	Retrospective cohort	N = 82 patients with CP-CML.	Baseline CKD in 12 patients, older age and lower eGFR at baseline	Imatinib 400 mg/day for a median duration of 105 months.	Statistically significant GFR decline after 5 years of imatinib therapy. ⇒ **New onset CKD, worsening of CKD, and renal anemia** ⇒ **Time to event: measured at 5 years**	Discontinuing imatinib in patients achieving durable molecular remission	Long-term use of imatinib can lead to reversible but progressive decline in eGFR. Decline in eGFR was correlated with anemia at 5 years due to lower EPO production.
	Teuma C, et al. (2016) *Full text* [23]	Case report	An 18-year-old man with CP-CML.	Not reported	Imatinib 400 mg daily.	Renal thrombosis 9 months post imatinib therapy secondary to glomerulopathy. Nephrotic syndrome with normal creatinine and eGFR. Biopsy: type I membranous glomerulonephritis with no malignant infiltration. ⇒ **Nephrotic syndrome (possibly secondary to glomerulonephritis)** ⇒ **Time to event: 9 months**	Holding imatinib. Anticoagulation. Prednisolone 1 mg/Kg/day.	Imatinib may induce nephrotic syndrome.
	Kitiyakara C, et al. (2002) *Full text* [20]	Case report	A 67-year-old man with BP-CML.	HTN, history of CKD, heart transplant cyclosporine therapy, blast crisis (TLS risk).	Cytarabine (Ara-C) + imatinib 600 mg daily.	AKI, superimposed on CKD: First event on day 4 of therapy: renal failure. **Imatinib was discontinued, and HD started for 4 days.** Ten days later, renal parameters improved, and **imatinib was restarted at same dose (600 mg/day) + Ara-C as blasts started to rise.** Second event on day 10 of restarting therapy: worsened renal function. Imatinib was stopped again. Eight days later, **imatinib was restarted at reduced dose (300 mg/day) + Ara-C as blasts started to rise again.** Third event on day 7 of restarting therapy:**imatinib was permanently discontinued.** Hemodialysis was resumed, and the patient became dialysis-dependent. Patient passed away 10 days after imatinib discontinuation. ⇒ **AKI, worsening of pre-existing CKD to dialysis** ⇒ **Time to event: 4 days**	Imatinib discontinuation and dose reduction. HD. Diuresis.	Imatinib-induced nephrotoxicity may recur upon rechallenging at same or lower doses. Patient passed away.
Imatinib	Rüžičková L, et al. (2015) *Conference abstract* [18]	Retrospective cohort	N = 48 patients with CP-CML (n = 47) and AP-CML (n = 1).	Baseline eGFR ≤ 60 mL/min/1.73 m^2^ were excluded, DM (n = 1), HTN (n = 6).	Imatinib (dose unspecified, for a median duration of 76.8 months).	Five patients developed AKI within the first month of imatinib therapy; effect was reversed in only two patients. Ten patients developed CKD (six had HTN, and one had DM). Treatment duration had a statistically significant correlation with eGFR decline. ⇒ **New onset AKI and New onset CKD** ⇒ **Time to event: AKI within first month of imatinib therapy, CKD (unspecified)**	Not specified	Long-term therapy with imatinib is correlated with declining eGFR.
	Breccia M, et al. (2017) *Conference abstract* [19]	Retrospective cohort	N = 320 patients with CP-CML over 9 years.	Not reported	Imatinib (dose unspecified).	Two patients died due to CKD. CKD was reported in 0.7% of the 255 patients who continued therapy. ⇒ **New onset CKD** ⇒ **Time to event: not reported**	Not specified	CKD may occur in imatinib-treated patients.
Dasatinib	Calizo R, et al. (2019) *Full text* [25]	Analysis of FAERS reports and in vitro study		Not reported	Dasatinib	High risk for nephropathies with dasatinib compared to other TKIs, especially glomerular events, but not tubular events. ⇒ **Nephrotic syndrome** ⇒ **Time to event: not reported**	Not specified	Dasatinib may induce nephrotoxicity, mostly nephrotic syndrome, through altered podocyte actin cytoskeleton.
	Loi V, et al. (2018) *Conference abstract* [26]	Case report	A 69-year-old woman with CP-CML	SAH,DM and dyslipidemia.	Hydroxyurea 1.5 g/day for 2 weeks, then dasatinib 100 mg daily	Eight months post dasatinib: significant deterioration in serum creatinine and nephrotic syndrome (hypoalbuminemia, proteinuria, edema, and mild pleural and pericardial effusions). Kidney biopsy: FSGS. All signs improved 6 months post stopping dasatinib and starting steroids. ⇒ **Nephrotic syndrome** ⇒ **Time to event: 8 months**	Stop dasatinib. Prednisone 0.5 mg/Kg/day	Dasatinib may induce nephrotic syndrome, possibly through FSGS.
Nilotinib	Ahn SY, et al. (2022) *Full text* [27]	Open-label mul-ticenter post-marketing surveillance study	N = 669 CML patients enrolled for safety analysis	Not reported	Newly diagnosed patients were all CP-CML = 328 patients—nilotinib 300 mg twice daily. Pre-treated patients with intolerance or resistance to prior therapies = 341 patients (nilotinib 400 mg/day); of those patients, CP-CML = 317 and AP-CML = 24.	Rare renal side effects were identified: AKI (0.1%). Renal disorder (undefined; 0.1%). Tubulointerstitial nephritis (0.1%). Ureteral disorder (0.1%).	Not specified	Renal ADRs are rare with nilotinib.
	Hua J, et al. (2013) *Full text* [28]	Case report	**Case #1**: 47-year-old man with CP-CML **Case #2**: 44-year-old man with CP-CML.	Case #1: renal parenchymal bleeding Case#2: not reported	**Case #1:** with hydroxyurea 2 g/day and nilotinib 600 mg daily. **Case#2:** treated with hydroxyurea 2 g/day, then nilotinib 150 mg daily.	**Case #1**: Four days after nilotinib initiation, patient had drastic drop in WBC count and developed TLS with AKI (resolved after holding nilotinib and did not recur after reinitiation at 200 mg/day and gradual titration to 600 mg/day over 20 days). **Case #2**: 9 h after nilotinib initiation, patient developed TLSand severe renal failure (metabolic acidosis) and continued to worsen with DIC and multiorgan failure and passed away 5 days after starting nilotinib despite supportive measures.	Case#1: Nilotinib held, then dose reduced.	Nilotinib titration should be considered instead of high-dose initiation to avoid development of TLS and associated renal complications. Monitoring and prevention of TLS with nilotinib should be considered.

SAH: Systemic Arterial Hypertension, CHF: Congestive Heart Failure, Hgb: Hemoglobin (g/dL), IHD: Ischemic Heart Disease, MI: Myocardial Infarction, BPH: Benign Prostatic Hyperplasia, N/A: not applicable, CV: cardiovascular, BP: Blast Phase, HD: hemodialysis, FSGS: Focal Segmental Glomerulosclerosis, PAD: Peripheral Arterial Disease, and EPO: erythropoietin.

#### 3.3.1. Imatinib

According to the identified studies, long-term use of imatinib was significantly associated with a progressive decline (usually reversible) in the estimated glomerular filtration rate (eGFR) [14,15,16,17,18]. AKI (time range 4 to 7 days) [18,20], new onset CKD (time range 1 to 5 years) [14,15,16,17,18,19], and deterioration of pre-existing kidney dysfunction (time range 4 days to 5 years) [16,17,20,21,22] were all frequently reported with imatinib use. Moreover, an imatinib-induced decline in eGFR was significantly correlated with a reduction in hemoglobin levels over time [15,16,17]. Three cases reported imatinib-induced progression of baseline CKD leading to dialysis initiation [20,21,22]. Of these three cases, two reported subsequent mortality [20,22], while one reported successful imatinib rechallenge using therapeutic drug monitoring (TDM) [21]. Renal vein thrombosis and subsequent nephrotic syndrome during imatinib therapy were also reported [23]. Overall, none of the reported imatinib-induced renal ADRs seemed to be dose-related (doses ranged between 100 mg and 800 mg). Conversely, no significant renal deterioration was observed in a CKD patient who underwent a second kidney transplant while on imatinib. In addition, renal ADRs were rarely reported in a 5.8-year retrospective cohort study of 320 patients, where only two fatalities were due to chronic renal failure and new onset CKD was reported in 0.7% of the 255 patients who remained on therapy [19,24].

#### 3.3.2. Dasatinib

Dasatinib was associated with a markedly high reporting of glomerular events, particularly nephrotic syndrome, compared to other TKIs in the FAERS analysis [25]. A case report also described dasatinib-induced glomerulosclerosis and nephrotic syndrome [26].

#### 3.3.3. Nilotinib

Renal events with nilotinib were rare in post-marketing data [27]. However, AKI was described as secondary to tumor lysis syndrome (TLS) in a report of two cases, where only one case survived and was successfully rechallenged with a slower nilotinib titration [28].

### 3.4. Renal Outcomes from Studies Reporting on Two TKIs

Nine studies reported renal outcomes with two TKIs, including seven case reports, one observational cohort study, and one extended analysis from three clinical trials [29,30,31,32,33,34,35,36,37]. Dasatinib was frequently identified as the culprit (n = 4 studies), followed by imatinib (n = 2). Comparisons included dasatinib versus nilotinib (n = 1) and imatinib versus bosutinib (n = 1) or dasatinib (n = 1). Table 6 describes the details of all the eligible studies in this section.

**Table 6:** Studies reporting renal events with two TKIs in CML patients.

Study		Design	Population	Comorbidities/Risk Factors	TKI	Renal Events	Interventions	Conclusion
Imatinib	Alshurafa A, et al. (2024) *Full text* [33]	Case report	A 62-year-old man with CML	DM, HTN, obesity, fatty liver, stage 3 CKD, and dyslipidemia	Imatinib 400 mg daily	Diuretic-resistant fluid retention on imatinib therapy causing periorbital edema, lower limb edema, weight gain, and shortness of breath, limiting activity. Imatinib was stopped, and asciminib 40 mg twice daily was started; led to maintenance of deep molecular remission and no worsening of renal function.	Diuretics Discontinuation of imatinib. Shifting to asciminib.	Imatinib-related edema may be worsened by pre-existing CKD. Shifting to asciminib with pre-existing CKD led to resolving edema, maintenance of efficacy, and no additional ADRs.
Imatinib	Gafter-Gvili A, et al. (2010) *Full text* [34]	Case report	A 60-year-old woman with CML	DM and HTN	Imatinib 400 mg daily	A gradual increase in creatinine levels from 0.94 mg/dL to 2.19 mg/dL over 9 months of imatinib therapy, accompanied by microalbuminuria progression. Imatinib was discontinued. Renal function normalized after 1 week, and patient was shifted to nilotinib 400 mg twice daily, after which renal parameters and efficacy outcomes remained stable at 3 months.	Discontinuation of imatinib and shifted to nilotinib after normalization of serum creatinine.	Imatinib-induced nephrotoxicity may be overcome by discontinuation and switching to nilotinib.
Dasatinib	TaniguchiY, et al. (2020) *Full text* [32]	Case report and pharmacokinetic study	A 64-year-old man with CML	Liver cirrhosis (Child-Pugh class A) and CKD on thrice weekly HD	Dasatinib 50 mg daily	HD elevated dasatinib levels led to severe pulmonary HTN and cardiotoxicity. Dasatinib was stopped, and bosutinib 200 mg daily was started. Bosutinib was partially removed by HD, so same dose was continued. Patient achieved target BCR-ABL at 3 months without reporting ADRs.	Stopping dasatinib and starting bosutinib.	Dasatinib with HD may lead to cardiotoxicity. TDM and cardiac function monitoring are recommended. Bosutinib may be safer in HD patients.
	Chang B.S. F, et al. (2019) *Conference abstract* [29]	Case report	A 48-year-old female with CP-CML	Not reported	Dasatinib (dose not reported)	Five months after starting dasatinib: proteinuria, AKI, and worsening HTN. Renal biopsy: renal TMA. Resolved after switching to imatinib.	Discontinuation of dasatinib and switching to imatinib.	Dasatinib may induce renal injury through TMA.
	Stanchina M, et al. (2020) *Full text* *Case 1* [31]	Case series. Only first case was included, as case 2 was B-ALL	A 53-year-old man with CML-AP	HTN	Mitoxantrone, high-dose cytarabine and **dasatinib 100 mg daily**	He developed scrotal and lower limb edema and proteinuria few days after treatment. Dasatinib was stopped, and imatinib was started (dose unspecified). Proteinuria improved within 3 days.	Stopping dasatinib and starting imatinib.	Dasatinib therapy may lead to edema and proteinuria that resolves by switching to imatinib.
	Uz B, et al. (2016) *Full text* [30]	Case report	A 52-year-old man with CP-CML	None and not on any medications	Dasatinib 100 mg daily resistant to imatinib therapy	TKI-induced rhabdomyolysis and AKI 2 weeks after starting dasatinib. CK elevation, myalgia, and AKI reduced after stopping dasatinib. Patient passed away due to severe pneumonia.	Stop dasatinib. Aggressive hydration. Allopurinol. Diuresis.	Dasatinib-induced AKI with myalgia may be related to rhabdomyolysis and should be monitored. Patient passed away.
Dasatinib	Cortes J, et al. (2015) *Conference abstract* [36]	Pooled trial analysis from three clinical trials	DASISION; N = 519 (newly diagnosed CP-CML) CA180-034 N = 670 (imatinib-resistant CP-CML) CA-180-035—excluded (ALL patients)	Not reported.	DASISION; treated with imatinib 400 mg/day or dasatinib 100 mg/day CA180-034: dasatinib TDD 100 mg/day or TDD 140 mg daily	DASISION: Imatinib: mean eGFR declined by 9 mL/min/1.73 m2 within the first 2 weeks of treatment and then stabilized. Dasatinib: minor increase in mean eGFR within the beginning of treatment, then returned to baseline levels. CA180-034: No difference in mean eGFR over time across all dasatinib doses.	Not specified	Dasatinib therapy did not affect renal function over time in those studies, compared to imatinib which led to developing CKD over time.
Imatinib								
Dasatinib	Mori J, et al. (2020) *Full text* [35]	Case report and pharmacokinetic study	A 66-year-old man with CML-CP	HTN and stage 1 CKD then HD.	Initially treated with nilotinib 300 mg twice daily. At 73 years old, he was shifted to dasatinib.	He developed nephrosclerosis during the 7 years of nilotinib therapy, so he started needing HD. Nilotinib was stopped, and dasatinib 100 mg daily was started with HD. Significant drop in EF (54% to 35%) occurred 6 months post dasatinib therapy, and TKI was stopped. Cardiac function did not recover. However, patient maintained molecular remission off-treatment.	Stopping TKI and attempting treatment-free remission	Nilotinib may lead to CKD progression to HD through nephrosclerosis. Dasatinib with HD may lead to cardiotoxicity; TDM and cardiac function monitoring are recommended.
Nilotinib								
Bosutinib	Cortes J, et al. (2017) *Full text* [37]	Retrospective analysis from two open-label studies	Study 1 (n = 546; n = 403 CP-CML, n = 79 AP-CML, n = 64 BP-CML). Additionally, ALL patients were excluded for the sake of this review. Study 2: CP-CML	Many had HTN, DM, and a small percentage had baseline CKD. eGFR was measured with MDRD equation.	Study 1: second-line or later bosutinib Study 2: Imatinib 400 mg/day (n = 251; median duration 49.5 months) or bosutinib 500 mg/day (n = 248; median duration 54.4 months).	**Study 1:** Thirteen percent developed renal ADRs with bosutinib second line or later. Of those patients, 19% had baseline renal events. Thirty-nine percent of those patients had reversible event with no subsequent worsening. **Study 2:** Bosutinib first line: 9% had renal events; none had baseline renal conditions. Reversible in 35% of the patients. Imatinib first line: 6% had renal events; none had baseline renal conditions. Reversible in 36% of the patients.	Holding TKI (most common). Dose reduction.	No major difference was observed between the imatinib-induced and bosutinib-induced decline in eGFR.
Imatinib								

TDD: Total Daily Dose, HD: hemodialysis, EF: ejection fraction, CK: Creatinine Kinase, ALL: Acute Lymphoblastic Leukemia, MDRD: Modification of Diet in Renal Disease.

#### 3.4.1. Dasatinib

Four of the case reports that described renal events on 2 TKIs related those ADRs to dasatinib [29,30,31,32]. The first case reported renal thrombotic microangiopathy (TMA) that presented with AKI, proteinuria and progressive HTN in a chronic phase (CP) CML. While a dasatinib dose was not provided, the renal event occurred following a dose escalation [29]. Baseline comorbidities were not described; however, the renal event resolved when dasatinib was switched to imatinib [29]. The second AKI case occurred in an imatinib-resistant CP-CML patient with no other comorbidities treated with dasatinib 100 mg daily [30]. The patient developed rhabdomyolysis that presented with myalgia, muscle weakness, and AKI 2 weeks following the start of dasatinib [30]. The event resolved with stopping dasatinib, hydration, diuresis, and allopurinol [30]. The patient passed away due to severe pneumonia and disease progression [30]. The third case described nephrotic syndrome in an acute-phase (AP) CML hypertensive patient treated with dasatinib 100 mg daily (*combined with chemotherapy*) [31]. The nephrotic syndrome occurred after starting therapy for a few days and resolved by stopping dasatinib and switching to imatinib therapy [31]. The last case described severe cardiopulmonary dasatinib-related ADRs in a hemodialysis cirrhotic CML patient [32]. The patient was switched from dasatinib 50 mg daily to bosutinib 200 mg daily, and no further ADRs were reported [32].

#### 3.4.2. Imatinib

Two cases reported renal ADRs that improved with switching to a different TKI [33]. The first case was in a patient with multiple baseline comorbidities, including stage 3 CKD [33]. The patient developed severe activity-limiting, diuretic-resistant edema with imatinib 400 mg daily that resolved with switching to asciminib 40 mg daily [33]. The second case involved AKI and microalbuminuria in a CML patient with baseline HTN and DM [34]. The patient was treated with imatinib 400 mg daily, and the renal ADR resolved after switching to nilotinib 400 mg twice daily [34].

#### 3.4.3. Nilotinib and Dasatinib

A case reported the incidence of worsening nephrosclerosis during the 7 years of nilotinib 300 mg twice daily therapy that led to the initiation of hemodialysis in a patient with baseline HTN, stage 1 CKD, and CP-CML [35]. Following the initiation of dialysis, the patient was switched to dasatinib 100 mg daily [35]. However, within 6 months, the patient’s cardiac function significantly deteriorated, leading to TKI discontinuation [35]. The patient remained in remission [35].

#### 3.4.4. Comparative Studies

The long-term analysis from dasatinib trials has shown that prolonged duration of dasatinib use did not cause eGFR decline nor new onset CKD, contrary to prolonged imatinib use [36]. In addition, bosutinib (both as first and second line) seemed to produce a time-dependent decline in eGFR comparable to imatinib therapy in a large retrospective analysis from two studies [37].

### 3.5. Renal Outcomes from Studies Reporting on Three or More TKIs

Eleven studies reported renal outcomes with at least three TKIs (four case reports and seven observational studies) [38,39,40,41,42,43,44,45,46,47,48]. Most of the studies reported imatinib use as the cause of renal events (n = 7 studies), followed by dasatinib (n = 2) and nilotinib (n = 1), while one study concluded on multiple TKIs. Table 7 describes the details of all the eligible studies in this section.

**Table 7:** Studies reporting renal events on at least three TKIs in CML patients.

Study		Design	Population	Comorbidities	TKIs Received	TKI(s) with Renal Incidents	Interventions
Nilotinib	Atef M, (2023) *Conference abstract* [47]	Case report.	A 22-year-old man with CP-CML.	Not reported.	○ Imatinib 400 mg daily (refractory at 6 months). ○ Nilotinib (dose unspecified; renal ADR; progression to BP). ○ Dasatinib (with chemotherapy; dose unspecified).	Nilotinib: Nephrotic syndrome (proteinuria; scrotal and lower limb edema). Renal biopsy: ○ No amyloid, kappa, lambda, C3, or immunoglobulin A or immunoglobulin G deposits. ○ Minimal changes due to CML and nephrotic syndrome.	Nilotinib discontinuation. Diuretics. Corticosteroids. Anticoagulation (apixaban). Statins.
Dasatinib	Holstein S, et al. (2008) *Full text* [45]	Case report.	A 58-year-old woman with AP-CML.	Not reported.	○ Imatinib 400 mg daily (progression to BP at 9 months). ○ Dasatinib 70 mg twice daily, then once daily due to cytopenia. Dose was increased again to twice daily due to disease progression (Renal ADR and HD, progression to BP). ○ Nilotinib 400 mg twice daily (disease progression and death).	Dasatinib: AKI (non-oliguric), pleural effusion, lower limb edema, hypoxia (with normal cardiac function). Then patient became anuric (no evidence of TLS), unresponsive to diuresis, and dependent on HD. Renal biopsy deferred.	Hydration (later stopped due to worsening pleural effusion). Diuretics (furosemide then mannitol). HD. Dasatinib discontinuation (due to disease progression, but renal function improved after starting nilotinib).
	Ong R, et al. (2014) *Conference abstract* [46]	Case report.	A 67-year-old patient with imatinib-resistant CML.	Not reported.	○ Imatinib 400 mg daily (dose unspecified, resistant). ○ Dasatinib (dose unspecified; renal ADR AKI and proteinuria).	Dasatinib: AKI and proteinuria. Renal biopsy: podocyte effacement. Improved after stopping dasatinib.	Stopping dasatinib.
Imatinib	Molica M, et al. (2018) *Full text* [38]	Retrospective cohort	N = 397 CP-CML patients.	Not reported; SCORE risk for CV was lowest among imatinib patients (high risk for CV events was 100% with nilotinib patients, 9.6% with imatinib patients and 33.4% with dasatinib patients). Excluded patients with baseline kidney dysfunction (eGFR < 60 mL/min), heart failure (NYHA class III-IV), poor performance status (ECOG >2), CML-AP or BP.	○ Imatinib (n = 320 patients); followed up for a mean duration of 9 years. Doses were 400 mg daily (n = 310 patients) and 300 mg daily (n = 10 patients). ○ Nilotinib (n = 53 patients); followed up for a mean duration of 2.5 years. Dose was 300 mg twice daily in all patients. ○ Dasatinib (n = 24 patients); followed up for a mean duration of 2.5 years. Dose was 100 mg in all patients.	* eGFR measured with the CKD-EPI equation Imatinib: Statistically significant decline in eGFR over time with 42 patients developing CV events without prior risk factors. Four percent had AKI in the first 12 months of therapy (median 12 days). An amount of 400 mg daily had no statistical difference in eGFR decline compared to 300 mg daily. **Nilotinib**: no decline in eGFR; one patient had ischemic event. No AKI was reported. **Dasatinib**: no decline in eGFR; three patients had ischemic event. AKI was reported in one patient in the first 12 months.	Not specified. However, authors mentioned that no patient had to discontinue any TKI due to AKI.
	Hino A, et al. (2016) *Full text* [39]	Retrospective cohort	N = 60 CP-CML patients.	Renal comorbidities (n = 5; CKD, IgA nephropathy, renal rupture due to RTA, Gitelman syndrome), chronic diseases (n = 9; DM, HTN, hyperlipidemia). No comorbidities (n = 32).	All initially treated with imatinib (dose unspecified) for a median duration of 101 months. Of those patients, 38 were changed to second-generation TKIs: ○ Nilotinib (n = 32 patients); for a median duration of 31 months. ○ Dasatinib (n = 6 patients); for a median duration of 37 months.	* eGFR measured using the three-variable Japanese equation. Imatinib: Statistically significant increase in serum creatinine and decline in eGFR from 1 year of therapy and continued over time. Twenty-five patients received prior lines of therapy (allogeneic HSCT, HU, IFN, busulfan and combinations of those agents). No statistical difference in eGFR decline between treatment-naïve and pre-treated patients. **Nilotinib and dasatinib (composite reporting)**: statistically significant improvement in eGFR, including patients who developed CKD.	Stopping imatinib and switching to second-generation TKI (dasatinib or nilotinib).
Imatinib	Yilmaz M, et al. (2015) *Full text* [44]	Retrospective cohort	N = 468 newly diagnosed CP-CML patients	Baseline HTN, DM, CV disease and CKD were similar at baseline between groups.	○ Imatinib (n = 253 patients; 400 mg twice daily in 207 patients and 400 mg once daily in 49 patients). ○ Nilotinib 400 mg twice daily (n = 116 patients). ○ Dasatinib 100 mg daily or 50 mg twice daily (n = 99 patients).	* eGFR measured with the MDRD equation AKI occurred in 19 patients (4%): Median time to AKI was 9 days (range 4 to 84 days). Of the 19 cases, 16 were on imatinib (15 on 800 mg/day and 1 on 400 mg/day), 2 were on nilotinib, and 1 was on dasatinib. Statistically significant association with older age and other comorbidities (DM, HTN, and CV diseases). New CKD occurred in 58 patients (14%): Median time to CKD was 12 months (range 3 to 108 months). Of the 58 cases, 49 were on imatinib (no difference between 400 and 800 mg/day), 4 were on nilotinib, and 5 were on dasatinib. Statistically significant association with older age and other comorbidities (DM, HTN, and CV diseases). Patients with CKD at baseline 48 patients: Mean eGFR significantly increased in the first 3 months with all TKIs. At the end of 24 months, eGFR slightly decreased with imatinib but increased slightly with dasatinib and drastically with nilotinib.	AKI:TKI held (4 of 19 patients) for a median of 10 days. CKD: eGFR in patients with pre-existing CKD may slowly improve during therapy with nilotinib and dasatinib.
	Breccia M, et al. (2017) *Conference abstract* [40]	Retrospective cohort	N = 397 newly diagnosed CP-CML patients	Not reported	○ Imatinib (n = 320 patients; dose unspecified; for median 8 years). ○ Nilotinib (n = 53 patients; dose unspecified; for median 2.5 years). ○ Dasatinib (n = 24 patients; dose unspecified; for median 2.5 years).	* eGFR measured with the CKD-EPI equation Imatinib: Significant decrease in eGFR over time (*p* = 0.01). Forty-two patients had cardiac events with significant eGFR decline over time (significant correlation; *p* = 0.04). Nilotinib: No decline in eGFR over time, and one patient had cardiac event unrelated to eGFR. Dasatinib: Statistically insignificant transient decline in eGFR that recovered spontaneously, and three patients had cardiac event without eGFR change.	Not mentioned
Imatinib	Ren X, et al. (2019) *Full text* [42]	Retrospective cohort.	N = 599 CP-CML patients over 16 years	Baseline CKD was excluded	○ **Cohort 1 (n = 460) patients on first-line TKI:** imatinib (n = 360), nilotinib (n = 100). ○ **Cohort 2 (n = 113) patients on second-line/third-line TKI:** nilotinib (n = 65), dasatinib (n = 74).	**Cohort 1:** Chronic renal adverse events (CRAE): 159 imatinib patients (44%; median 12 months) vs. 20 nilotinib patients (20%; median 21 months). CKD: 82 imatinib patients (23%; median 12 months) vs. 8 nilotinib patients (8%; median 6 months). CRAE-free and CKD-free survival were significantly lower with imatinib compared with nilotinib (*p* = 0.001 and 0.021, respectively). Older age, male gender, previous non-TKI therapies and longer time from diagnosis to TKI initiation were significant risk factors for lower CRAE survival. Fifty-five patients underwent monitoring for glomerular/renal tubular function. At 6 months, 24 h protein levels were significantly increased with imatinib without significant changes with nilotinib. **Cohort 2:** CRAE: 9 nilotinib patients (14%; median 24 months) vs. 13 dasatinib (18%; median 18 months). CKD: 5 nilotinib patients (8%; median time 30 months) vs. 5 dasatinib patients (7%; median time 18 months). There were no significant differences in CRAE-free and CKD-free survival between nilotinib and dasatinib. Previous non-TKI therapies was the only significant risk factor for lower CRAE survival. Patients switching from imatinib to nilotinib (8)/dasatinib (n = 18) due to progression (n = 27) without kidney-relayed toxicities: eGFR increased significantly at 6 months with both agents.	Not mentioned.
Imatinib	Sönmez Ö, et al. (2024) *Full text* [41]	Retrospective cohort.	**Cohort 1**: 195 patients treated with upfront imatinib **Cohort 2 (control group)**: 138 CKD patients.	9.2% in CML group had DM, and 19.5% had HTN.	**Cohort 1**: 195 patients treated with upfront imatinib (400 mg/day for all patients except for two who were on 300 mg/day). However, at the time of analysis: **Patients** still on imatinib; n = 131 ○ Nilotinib (n = 15) ○ Dasatinib (n = 16) ○ Bosutinib (n = 2) ○ Ponatinib (n = 2)	Imatinib patients: Three developed AKI, two of them within the first month of therapy. Twenty-one patients developed new CKD (risk factor: lower baseline eGFR; *p* < 0.001). eGFR declined by 1.751 per year in imatinib group (not statistically significant) compared to 1.872 per year in CKD group. No statistically significant eGFR decline with second-generation TKIs, but patients on bosutinib had a downward trend.	Holding imatinib.
	Costa A, et al. (2024) *Full text* [43]	Retrospective cohort	Newly diagnosed CML patients who are at least 75 years old (N = 123); median age 80 years old.	Cardiovascular disease (82.1%), COPD (10.5%), CKD (9.7%), dyslipidemia (34.9%), DM (10.4%), BPH (16.2%), and other cancer history (17.8%).	First-line (123): Imatinib (n = 106) Nilotinib (n = 7) Dasatinib (n = 9) Bosutinib (n = 1) Thirty-one patients switched to second-line therapy with: Imatinib (n = 1) Nilotinib (n = 6) Dasatinib (n = 13) Bosutinib (n = 9) Ponatinib (n = 2) Nine patients switched to third-line therapy with: Nilotinib (n = 2) Dasatinib (n = 1) Bosutinib (n = 1) Ponatinib (n = 5) One patient switched to fourth-line therapy with asciminib.	General ADRs were reported in 97 patients. **Renal events were only reported with imatinib (no statistical calculation):** AKI in 1.22% of reported ADRs. Increased serum creatinine in 0.61% of ADRs. CKD in 0.61%.	No specific interventions for renal ADRs were highlighted.
Multiple	Uchida Y, et al. (2024) *Full text* [48]	Case report	A 63-year-old male patient with CP-CML	History of kidney transplant (on long-term prednisone, tacrolimus, mycophenolate mofetil).	Imatinib 400 mg/day. Dasatinib 20 mg/day. Nilotinib 150 mg/day, then 300 mg/day. Asciminib 40 mg/day.	**Imatinib (400 mg/day)** Oliguria, weight gain, and drop in eGFR from 26 to 11 within 1 week. **Dasatinib (20 mg/day)** Diarrhea, oliguria, and weight gain within 1 day. **Nilotinib (150 mg/day, then 200 mg/day, then 300 mg/day)** Renal function deteriorated after increasing the dose. **Asciminib (40 mg/day)** No renal events and undetectable BCR::ABL1 with concerns for resistance.	Discontinuing offending TKI and switching to another while being mindful of renal dosage.

BP: Blast Phase, HD: hemodialysis, NYHA: New York Heart Association, ECOG: Eastern Cooperative Oncology Group, CV: cardiovascular, SCORE: Systematic Coronary Risk Evaluation, RTA: Road Traffic Accident, HSCT: hematopoietic stem cell transplant, HU: hydroxyurea, IFN: interferon-alpha, MDRD: Modification of Diet in Renal Disease, CKD-EPI: Chronic Kidney Disease EPIdemiology collaboration, COPD: chronic obstructive pulmonary disease, BPH: benign prostatic hyperplasia.

#### 3.5.1. Imatinib

Three retrospective studies have shown a statistically significant time-dependent decline in eGFR with imatinib when compared with dasatinib and nilotinib [38,39,40]. In fact, the decline in eGFR over time with imatinib was comparable to that of CKD (non-CML) patients in a fourth retrospective study [41]. It also showed no significant eGFR decline with nilotinib, dasatinib, or bosutinib, but the latter had a downward trend in eGFR [41]. In addition, when compared to nilotinib, imatinib therapy was not only linked to more renal events but also significantly lower renal event-free survival [42]. On the other hand, there was no significant difference between nilotinib and dasatinib therapy [42]. Furthermore, a retrospective cohort study involving multiple TKIs focused on patients who are at least 75 years old did not report renal ADRs with any medications other than imatinib. [43]. The higher likelihood of renal events with imatinib was also associated, with statistical significance, with multiple risk factors, including older age, comorbidities (DM, HTN, and coronary artery disease), and longer duration of TKI therapy [41,44].

#### 3.5.2. Dasatinib

Two cases described varying severities of AKI with dasatinib therapy in imatinib-resistant CML patients [45,46]. The first case described recurrent dasatinib-induced AKI, edema, pleural effusion, and pancytopenia in a blast-phase CML patient (*comorbidities unspecified*) [45]. The patient experienced these side effects with dasatinib 70 mg twice daily, once daily, and every other day [45]. The renal impairment continued to worsen to the point of regular hemodialysis [45]. Later on, the patient progressed on dasatinib and was shifted to nilotinib 400 mg twice daily, and urine output improved [45]. However, the patient passed away a month after starting nilotinib due to disease progression [45]. The second case described dasatinib-induced (*dose unspecified*) AKI and proteinuria in an imatinib-resistant CML patient [46]. The patient’s comorbidities were not specified; however, renal biopsy showed podocyte effacement, and the patient improved after stopping dasatinib [46].

#### 3.5.3. Nilotinib

In a patient with imatinib-resistant CP-CML [*comorbidities unspecified*], nephrotic syndrome occurred during nilotinib therapy [*dose unspecified*] [47]. However, renal biopsy showed minimal changes due to CML and nephrotic syndrome [47]. The patient improved with the discontinuation of nilotinib and initiation of diuretics, statins, corticosteroids and anticoagulation [apixaban] [47]. Later on, the patient progressed to blast crisis and improved with dasatinib and chemotherapy [47].

#### 3.5.4. Multiple TKIs

A case report described renal events with three TKIs but not with asciminib [48]. The patient had a history of renal transplant and was maintained on immunosuppressants [prednisone, tacrolimus, and mycophenolate mofetil] [48]. The patient still had CKD at the time of therapy initiation [eGFR 26 mL/min/1.73 m^2^] [48]. He developed oliguria, weight gain, and a drop in eGFR within a week of starting imatinib 400 mg/day [48]. The oliguria and weight gain recurred with dasatinib 20 mg/day [48]. With nilotinib therapy, the patient tolerated 150 mg/day and 200 mg/day; however, his eGFR started to decline with 300 mg/day [48]. The patient was switched to asciminib 40 mg and achieved remission as well as no further renal events [48].

### 3.6. Risk of Bias Assessment of Evidence

The JBI tool was used to assess the quality of the evidence of case reports (Table 8) and observational studies (Table 9). Generally, the reporting quality of all the included case reports (Table 8) was satisfactory. Four cases lacked adequate descriptions of the patient’s characteristics and TKI dosage. Although not all studies presented the case timeline through a figure, if the text description was detailed, this item was considered satisfactory.

As for observational studies (Table 9), most of the studies failed to report how confounders were accounted for but reported the ADRs in a satisfactory manner. In addition, a major concern was the significantly longer duration of follow-up with imatinib compared to other groups [41,42,44], the presence of significant confounders in one group over the other [41], as well as incomplete reporting of methodology.

However, the quality of FAERS analyses and post-marketing surveillance studies could not be assessed due to limitations in baseline information. Similarly, the risk of bias could not be performed for the long-term analysis from clinical trials [36]. A major concern in this analysis is that confounders were not identified nor analyzed in this subgroup.

### 3.7. Overall Recommendations

Based on the evidence presented, recommendations were derived from the studies reporting renal events with TKIs and compiled into the table below (Table 10).

**Figure 2:**
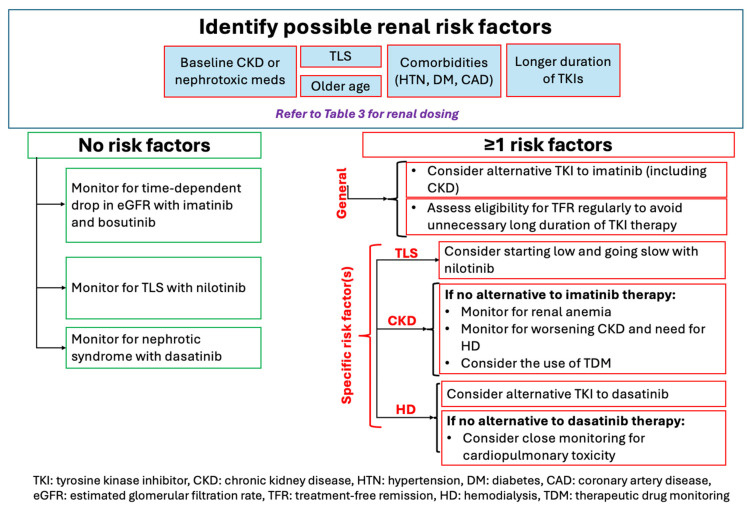
Suggestions on the initial choice of TKI in CML therapy and related monitoring parameters based on renal risk factors.

**Figure 3:**
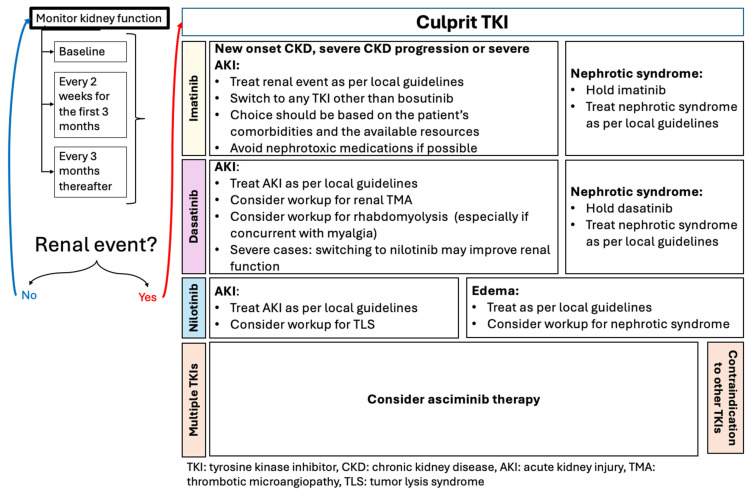
Suggestions on patient management following the occurrence of a TKI-related renal event.

The findings from all the previous sections were compiled into usable algorithms that may provide clinicians with suggestions to consider when navigating renal outcomes in CML patients (Figure 2 and Figure 3). It is crucial to highlight that further evidence is needed to evaluate whether the use of these suggestions may improve renal outcomes in CML patients receiving TKI therapy.

## 4. Discussion

This is the first review to systematically summarize the literature surrounding the incidence of renal events with TKI therapy in patients with CML. Additionally, it is the first review to offer practical recommendations for the selection of TKIs to mitigate renal ADRs. Although the mechanism of nephrotoxicity has not yet been established, it is thought to be due to both on- (oncoprotein BCR-ABL) [61] and off-target effects [44]. Off-target effects that could lead to renal damage include Platelet-Derived Growth Factor Receptor (PDGFR), c-KIT, SRC, and Vascular Endothelial Growth Factor (VEGF) [62]. These off-target inhibitions may lead to podocyte and renal tubular damage [62].

Renal events are still considered relatively rare with TKI therapy in CML. In an analysis of renal events from Vigibase (the World Health Orgnization global safety database), renal ADRs reported with BCR::ABL TKIs were analyzed [62]. A total of 1409 cases were reported over 20 years for imatinib, bosutinib, dasatinib, nilotinib, and ponatinib [62]. Around 50% of the reports were related to imatinib; both nilotinib and dasatinib were related to nephrotic syndrome, while nilotinib and ponatinib were related to renal artery stenosis [62]. The frequency of renal events with imatinib and nephrotic syndrome with dasatinib was consistent with the findings here from the primary literature [63]. However, none of the major renal events identified in this review mentioned renal artery stenosis with ponatinib. Nephrotic syndrome and renal artery stenosis were rarely mentioned with nilotinib in the primary literature.

Despite this rarity of renal impairment, its presence can increase the incidence of cardiovascular events, for which many TKIs are considered a risk factor [6,7,34,64]. In fact, the low incidence of renal events reflects on the design of the available literature, as most of it consisted of case reports and observational studies that were presented as conference abstracts. Although these factors limited the quality of the included studies, these events were not prevalent to show in large clinical trials, which makes it unfeasible to await trial-reported management of TKI-induced nephrotoxicity. Furthermore, the quality of reporting for most of the included studies was sufficient. The choice of the JBI tool for quality assessment was mainly due to it being one of the few tools that assess the quality of case reports. In addition, the use of JBI instead of the CASP tool for the quality assessment of observational studies was for standardization purposes to avoid discrepancies if multiple tools were used.

This review has emphasized the significant time-dependent decline in eGFR with imatinib therapy from the literature. However, it is important to be mindful of the difference in duration of market exposure, with imatinib being the first prototype compared to newer TKIs. Moreover, the current literature is subjective regarding the most appropriate sequence of TKI use in cases of toxicity. From the evidence presented herein, bosutinib also appears to cause a downward trend in eGFR, although statistically insignificant [37,41]. In addition, dasatinib-related cardiopulmonary toxicities appeared to be more severe in patients on hemodialysis [32,35]. Therefore, in the case of an imatinib-related decline in eGFR, bosutinib and dasatinib may not be the optimal subsequent therapies if the patient becomes dialysis-dependent. What is more is that both dasatinib (non-dialysis patients) and nilotinib, in particular, improved renal function following imatinib-induced nephrotoxicity [39,42,44], which makes them reasonable alternatives in the case of imatinib-related nephrotoxicity. In contrast, changing from dasatinib to imatinib in the case of nephrotic syndrome and TMA induced by the former led to alleviation of the renal event [29,31].

As such, despite the evidence being built by observational data, it allowed for designing a general scheme for tackling TKI-related nephrotoxicity in CML patients. However, the management of the actual event (AKI, nephrotic syndrome, and CKD) depended on local practices in each study. Therefore, providing a standardized approach apart from TKI choice is challenging with the current evidence.

This review highlights the need for individualized patient assessment for treatment-free remission (TFR) eligibility. This is because imatinib-related eGFR decline and new onset CKD appeared to be linked to a longer duration of use. Perhaps the increased utilization of TFR, when appropriate [65], may lead to reduced incidence. In addition, drug–drug and drug–disease interactions, particularly with DM and HTN, appeared to be important factors to consider in the prevention of TKI-related nephrotoxicity [44], thereby emphasizing the need for a holistic approach upon prescribing TKIs to CML patients.

## 5. Conclusions

This systematic review is the first to comprehensively identify and summarize renal ADRs associated with TKIs in CML, paving the way for a practical framework to manage renal impairment in these patients. Overall, imatinib seemed to be significantly linked to renal events (particularly CKD incidence) compared to other TKIs and switching to dasatinib or nilotinib significantly improved renal function. Moreover, bosutinib seems to be following the path of imatinib, although without statistical significance. While dasatinib had no effect on eGFR decline, it seemed more associated with nephrotic syndrome and a higher incidence of cardiopulmonary toxicity in hemodialysis patients. Lastly, nilotinib was observed to rarely cause renal ADRs through TLS, nephrotic syndrome, and nephrosclerosis. In addition, this review flagged the need for constant vigilance for renal events among CML patients on TKIs. High-quality reporting of these ADRs in the literature, especially with newer TKIs, is vital to build on the evidence presented herein. Future research should focus on several key areas, including long-term, large-scale studies to assess the chronic effects of TKIs on renal function, exploring the mechanism of TKIs inducing renal ADRs and investigating pharmacogenomics to personalize treatment and predict renal complications. Moreover, updating the clinical guidelines based on emerging evidence is crucial. Finally, fostering multidisciplinary collaboration among hematologists, nephrologists, and pharmacists is essential for advancing personalized treatment strategies and improving the overall management in this population.

## Figures and Tables

**Table 1:** National Cancer Institute CTCAE (CTCAE version 5.0) Grading Severity of Renal ADRs.

Grade	1	2	3	4	5
AKI	N/A	N/A	Hospitalization	Life-threatening/dialysis	Death
CKD	eGFR/CrCl = 60 to < LLN OR proteinuria 2+ present; urine protein/creatinine > 0.5	eGFR/CrCl = 30 to 59	eGFR/CrCl = 15 to 29	eGFR or CrCl < 15; dialysis OR renal transplant indicated	Death

LLN: Lower Limit for Normal; eGFR/CrCl measured in mL/min.

**Table 2:** Renal and urinary ADRs reported with CML TKIs according to FAERS from inception to 30 June 2023.

FDA-Approved TKI (Approval Date)	Number of ADRs	Number of Related Fatalities
Imatinib * (October 2001)	1009	268
Dasatinib ± (June 2006)	299	29
Nilotinib # (October 2007)	636	142
Bosutinib § (September 2012)	173	17
Ponatinib $ (December 2012)	573	118
Asciminb + (October 2021)	25	2

Results were filtered to “chronic myeloid leukemia as reason for use”, “renal and urinary disorders as reaction group”, and suspect ingredients were limited to * Imatinib or imatinib mesylate only, ± dasatinib or dasatinib anhydrous only, # nilotinib or nilotinib hydrochloride anhydrous or nilotinib hydrochloride mohohydrate only, § bosutinib or bosutinib monohydrate only, $ ponatinib only, and + asciminib or asciminib hydrochloride only.

**Table 8:** Risk of Bias Assessment: Cases Reporting Renal ADRs TKIs in CML patients.

Criteria	IM							DAS							NIL		DAS	NIL	+
	Nakahara [21]	Mestroni [22]	Thiem [24]	Teuma [23]	Kitiyakara [20]	Alshurafa [33]	Gafter-Gvili [34]	Loi [26]	Taniguchi [32]	Chang [29]	Stanchina [31]	Uz [30]	Holstein [45]	Ong [46]	Hua [28]	Atef [47]	Mori [35]		Uchida [48]
Were patient’s demographic characteristics clearly described?	Y	Y	Y	N	Y	Y	Y	Y	Y	N	Y	Y	N	N	Y	N	Y		Y
Was the patient’s history clearly described and presented as a timeline?	Y	Y	Y	Y	Y	Y	Y	Y	Y	Y	N	Y	Y	N	Y	Y	N		Y
Was the current clinical condition of the patient on presentation clearly described?	Y	Y	Y	Y	Y	Y	Y	Y	Y	Y	Y	Y	Y	Y	Y	Y	Y		Y
Were diagnostic tests or assessment methods and the results clearly described?	Y	Y	Y	Y	Y	Y	Y	Y	Y	Y	Y	Y	Y	Y	Y	Y	Y		Y
Was the intervention(s) or treatment procedure(s) clearly described?	Y	Y	Y	Y	Y	Y	Y	Y	Y	N	Y	Y	Y	N	Y	N	Y		Y
Was the post-intervention clinical condition clearly described?	Y	Y	Y	Y	Y	Y	Y	Y	Y	Y	Y	Y	Y	Y	Y	Y	Y		Y
Were adverse events (harms) or unanticipated events identified and described?	Y	Y	Y	Y	Y	Y	Y	Y	Y	Y	Y	Y	Y	Y	Y	Y	Y		Y
Does the case report provide takeaway lessons?	Y	Y	Y	Y	Y	Y	Y	Y	Y	Y	Y	Y	Y	Y	Y	Y	Y		Y

Abbreviations: IM: imatinib, DAS: dasatinib, NIL: nilotinib, Y: yes (green), N: no (red), +: more than two TKIs.

**Table 9:** Risk of bias assessment: Observational studies reporting renal ADR TKIs in CML patients.

Criteria	IM													IM	BOS
	Celestino [14]	Singh [15]	Moura [16]	Sakurai [17]	Rüžičková [18]	Breccia [19]	Molica [38]	Hino [39]	Yilmaz [44]	Breccia [40]	Ren [42]	Sönmez [41]	Costa [43]	Cortes [37]	
Were the two groups similar and recruited from the same population?	NA	NA	NA	NA	NA	NA	Y	NA	N	UC	N	N	NA	N	
Were the exposures measured similarly to assign people to both exposed and unexposed groups?	NA	NA	NA	NA	NA	NA	Y	NA	Y	UC	Y	NA	NA	Y	
Was the exposure measured in a valid and reliable way?	UC	UC	Y	Y	N	UC	Y	Y	Y	UC	Y	Y	Y	Y	
Were confounding factors identified?	Y	UC	Y	N	Y	UC	Y	Y	Y	UC	Y	Y	Y	Y	
Were strategies to deal with confounding factors stated?	UC	UC	Y	N	Y	UC	N	Y	Y	UC	Y	Y	UC	Y	
Were the groups/participants free of the outcome at the start of this study (or at the moment of exposure)?	UC	UC	N	N	Y	UC	Y	N	Y	Y	Y	N	N	N	
Were the outcomes measured in a valid and reliable way?	UC	Y	Y	Y	Y	Y	Y	Y	Y	Y	Y	Y	Y	Y	
Was the follow-up time reported and sufficient to be long enough for outcomes to occur?	Y	Y	Y	Y	Y	Y	Y	Y	Y	Y	Y	Y	Y	Y	
Was follow-up complete, and if not, were the reasons for loss to follow-up described and explored?	UC	Y	Y	UC	Y	Y	Y	Y	Y	UC	Y	Y	Y	Y	
Were strategies to address incomplete follow-up utilized?	UC	NA	N	UC	NA	NA	N	N	Y	UC	N	N	N	Y	
Was appropriate statistical analysis used?	Y	Y	Y	UC	UC	Y	Y	UC	Y	UC	Y	Y	Y	Y	

Abbreviations: IM: imatinib, Y: yes, (green) N: no (red), UC: unclear (bright yellow), NA: not applicable (dark gray).

**Table 10:** Recommendations from the studies on CML patients reporting renal outcomes on TKIs.

Clinical Scenario		Recommendations		
Pre-existing risk factors (older age [>60 years old], comorbidities, or lower eGFR at baseline)		Consider alternative TKI to imatinib Consider monitoring for time-dependent decline in eGFR with imatinib therapy		
Baseline CKD		Consider alternative TKI to imatinib		
In patients on baseline hemodialysis		Consider alternative TKI to dasatinib		
Renal events while on imatinib therapy		Consider switching to dasatinib or nilotinib if no contraindications Avoid bosutinib while taking into consideration the patient’s comorbidities and the available resources		
AKI on nilotinib therapy		Consider starting at a lower dose and titrating therapy Monitor for TLS and related renal impairment Consider workup for nephrotic syndrome if edema		
AKI on dasatinib therapy		Monitor for nephrotic syndrome during therapy Consider workup for renal TMA (especially in patients presenting with concurrent proteinuria and progressive HTN) Consider workup for rhabdomyolysis (particularly in patients with concurrent myalgia) Consider switching to nilotinib		
If imatinib therapy cannot be avoided		Monitor for superimposed AKI and progression of CKD leading to dialysis Monitor for CKD-related anemia with long treatment durations Avoid concomitant nephrotoxic medications with imatinib Consider the use of TDM with imatinib therapy if available		
If dasatinib therapy cannot be avoided		Consider close monitoring for cardiopulmonary ADRs		
Renal events despite switching TKIs		Consider asciminib therapy In patients with prolonged remission on TKI therapy: ○ Consider weighing the risk vs. benefit of continuing TKI therapy ○ Consider attempting the discontinuation of TKI and pursuing treatment-free remission to reduce time-dependent renal ADRs		
Patients with the below risk factors may be at higher risk of developing TKI-induced renal events:				
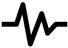	Diabetes		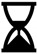	Long-term use of TKIs
	Hypertension			
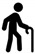	Older age		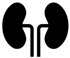	Baseline CKD or hemodialysis
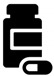	Concomitant use of nephrotoxic medications		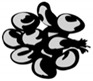	TLS

eGFR: Estimated glomerular filtration rate, AKI: Acute kidney injury, TKI: Tyrosin kinase inhibitor, CKD: Chronic kidney disease, TDM: Therapeutic drug monitoring, TLS: Tumor lysis syndrome, TMA: Thrombotic microangiopathies, HTN: Hypertension, and ADRs: Adverse drug reactions.

## Data Availability

The data that support the findings of this study are available on request from the corresponding author(s) M.B. and M.Y.

## Supplementary Materials

The following supporting information can be downloaded at: https://www.mdpi.com/article/10.3390/cancers17010092/s1, Text S1: Detailed search strategy per database; Table S2: Excluded studies and reasons for exclusion.
