# Supplementary material for: Caught in the Crossfire: Unmasking the Silent Renal Threats of Tyrosine Kinase Inhibitors in Chronic Myeloid Leukemia
Source: Cancers (Basel). 2024 Dec 30;17(1):92. doi: 10.3390/cancers17010092 (PMC11720497; doi:10.3390/cancers17010092)
Supplement: Supplementary file 1 [file cancers-17-00092-s001.zip › cancers-3337638-supplementary.pdf]

Renal ADRs with TKIs: Supplementary Material

Appendix S1: Detailed search strategy per database.

PubMed

(("imatinib mesylate"[MeSH Terms] OR ("imatinib"[All Fields] AND "mesylate"[All Fields]) OR "imatinib mesylate"[All Fields] OR "imatinib"[All Fields] OR "imatinib s"[All Fields] OR ("dasatinib"[MeSH Terms] OR "dasatinib"[All Fields]) OR ("asciminib"[Supplementary Concept] OR "asciminib"[All Fields]) OR ("ponatinib"[Supplementary Concept] OR "ponatinib"[All Fields]) OR ("nilotinib"[Supplementary Concept] OR "nilotinib"[All Fields]) OR ("bosutinib"[Supplementary Concept] OR "bosutinib"[All Fields])) AND "renal insufficiency"[All Fields]) AND ((fft[Filter]) AND (humans[Filter])).

EMBASE

'chronic myeloid leukemia'/exp AND ('chronic kidney failure'/exp OR 'acute kidney failure'/exp) AND ('imatinib'/exp OR 'dasatinib'/exp OR 'nilotinib'/exp OR 'ponatinib'/exp OR 'asciminib'/exp OR 'bosutinib'/exp) AND [embase]/lim NOT ([embase]/lim AND [medline]/lim) AND 'human'/de.

Table S2: Excluded studies and reasons for exclusion.

N	Source	Screening phase	Study	Reason for exclusion
1	EMBASE	Title	Uramatsu T, et al. 2017	Not CML treated with TKI and renal outcomes
2	EMBASE	Title	Sekhon B.S., et al. 2010	Not CML treated with TKI and renal outcomes
3	EMBASE	Title	Maroz N., et al. 2014	Not CML treated with TKI and renal outcomes
4	EMBASE	Title	Maiocco G., et al. 2023	Not CML treated with TKI and renal outcomes
5	EMBASE	Title	Varadarajan I., et al. 2015	Not CML treated with TKI and renal outcomes
6	EMBASE	Title	Haroon A., et al. 2020	Not CML treated with TKI and renal outcomes
7	EMBASE	Title	Onuigbo M.A.C., et al. 2020	Not CML treated with TKI and renal outcomes
8	EMBASE	Title	Yazdanie N., et al. 2018	Not CML treated with TKI and renal outcomes
9	EMBASE	Title	Karla P., et al. 2021	Not CML treated with TKI and renal outcomes
10	EMBASE	Title	Costa A., et al. 2012	Not CML treated with TKI and renal outcomes
11	EMBASE	Title	Keraliya A.R., et al. 2015	Not CML treated with TKI and renal outcomes
12	EMBASE	Title	Tobon A., et al. 2020	Not CML treated with TKI and renal outcomes
13	EMBASE	Title	D’Addona M., et al. 2022	Not CML treated with TKI and renal outcomes
14	EMBASE	Title	Singh S., et al. 2021	Renal outcomes are unclear
15	EMBASE	Title	Pal C.A., et al. 2023	Baseline renal impairment improved with TKI
16	EMBASE	Title	Seiter K.P., et al. 2011	Not CML treated with TKI and renal outcomes
17	EMBASE	Title	Kanstrup Fiehn A., et al. 2011	Not CML treated with TKI and renal outcomes
18	EMBASE	Title	Masroujeh R., et al. 2018	Not CML treated with TKI and renal outcomes
19	EMBASE	Title	Funasaka C., et al. 2019	Not CML treated with TKI and renal outcomes

20	EMBASE	Title	Farrer F., et al. 2010	Not CML treated with TKI and renal outcomes
21	EMBASE	Title	Kumar A., et al. 2018	Not CML treated with TKI and renal outcomes
22	EMBASE	Title	Tavitian S., et al. 2017	Not CML treated with TKI and renal outcomes
23	EMBASE	Title	Sherwood G.B., et al. 2016	Not CML treated with TKI and renal outcomes
24	EMBASE	Title	Bull T., et al. 2021	Not CML treated with TKI and renal outcomes
25	EMBASE	Title	Turrini M., et al. 2014	Not CML treated with TKI and renal outcomes
26	EMBASE	Title	Smith M.H., et al. 2013	Not CML treated with TKI and renal outcomes
27	EMBASE	Title	Yuan J., et al. 2023	Not CML treated with TKI and renal outcomes
28	EMBASE	Title	Apperley J.F., et al. 2021	Not CML treated with TKI and renal outcomes
29	EMBASE	Title	Khan S., et al. 2020	Not CML treated with TKI and renal outcomes
30	EMBASE	Title	Gerbouin O., et al. 2009	Not CML treated with TKI and renal outcomes
31	EMBASE	Title	Dudzisz-śledź M., et al. 2022	Not CML treated with TKI and renal outcomes
32	PUBMED	Title	Chen X., et al. 2024	Not CML treated with TKI and renal outcomes
33	PUBMED	Title	Kim SR., et al. 2021	Not CML treated with TKI and renal outcomes
34	PUBMED	Title	Fujii T., et al. 2020	Not CML treated with TKI and renal outcomes
35	PUBMED	Title	Bonse J., et al. 2018	Not CML treated with TKI and renal outcomes
36	PUBMED	Title	Zhuang Q., et al. 2015	Not CML treated with TKI and renal outcomes
37	PUBMED	Title	Skartsis N., et al. 2014	Not CML treated with TKI and renal outcomes
38	PUBMED	Title	Kay J., et al. 2008	Not CML treated with TKI and renal outcomes
39	PUBMED	Title	Shin K., et al. 2008	Not CML treated with TKI and renal outcomes
40	PUBMED	Title	Sonmez M., et al. 2008	Not CML treated with TKI and renal outcomes
41	PUBMED	Title	Pollack IF., et al. 2007	Not CML treated with TKI and renal outcomes
42	PUBMED	Title	Vuky J., et al. 2006	Not CML treated with TKI and renal outcomes
43	EMBASE	Abstract	O'Brien S., et al. 2019	Not CML treated with TKI and renal outcomes
44	EMBASE	Abstract	Rea D., et al. 2021	Ineligible publication type (Book chapter)
45	EMBASE	Abstract	Abaza Y., et al. 2016	Not CML treated with TKI and renal outcomes
46	EMBASE	Abstract	Pagnano K.B.B., et al. 2015	Not CML treated with TKI and renal outcomes
47	EMBASE	Abstract	Pagnano K.B.B., et al. 2021	Not CML treated with TKI and renal outcomes
48	EMBASE	Abstract	Wang X.A., et al. 2021	Not CML treated with TKI and renal outcomes
49	EMBASE	Abstract	Choi J., et al. 2018	Not CML treated with TKI and renal outcomes

50	EMBASE	Abstract	Samad R., et al. 2015	Not CML treated with TKI and renal outcomes
51	EMBASE	Abstract	Nunes R.A.B., et al. 2023	Not CML treated with TKI and renal outcomes
52	EMBASE	Abstract	Yılmaz U., et al. 2022	Not CML treated with TKI and renal outcomes
53	EMBASE	Abstract	Eghtedar A., et al. 2010	Not CML treated with TKI and renal outcomes
54	EMBASE	Abstract	Sincan G., et al. 2022	Not CML treated with TKI and renal outcomes
55	EMBASE	Abstract	Rivera-Torres J., et al. 2019	Not CML treated with TKI and renal outcomes
56	EMBASE	Abstract	Malyszko J., et al. 2019	Ineligible publication type (Book chapter)
57	EMBASE	Abstract	Aoyama R., et al. 2016	Not CML treated with TKI and renal outcomes
58	EMBASE	Abstract	Olavarria E., et al. 2013	Not CML treated with TKI and renal outcomes
59	PUBMED	Abstract	Tokumoto T., et al. 2023	No renal outcomes with TKI
60	EMBASE	Abstract	Barta V.S., et al. 2017	Not CML treated with TKI and renal outcomes
61	EMBASE	Full-text	Lovell A., et al. 2021	Ineligible publication type (Book chapter)
62	PUBMED	Full-text	Levêque D., et al. 2020	Not CML treated with TKI and renal outcomes
63	PUBMED	Full-text	Moore AE., et al. 2012	Not CML treated with TKI and renal outcomes
64	EMBASE	Full-text	Okayama Y., et al. 2021	Not CML treated with TKI and renal outcomes
65	EMBASE	Full-text	Castagnetti F., et al. 2021	Not CML treated with TKI and renal outcomes
66	EMBASE	Full-text	Bonifacio M., et al. 2019	Not CML treated with TKI and renal outcomes
67	EMBASE	Full-text	Capodanno I., et al. 2017	Not CML treated with TKI and renal outcomes
68	EMBASE	Full-text	Hughes T., et al. 2020	Not CML treated with TKI and renal outcomes
69	EMBASE	Full-text	Seth T., et al. 2011	Not CML treated with TKI and renal outcomes
70	EMBASE	Full-text	Hughes T.P., et al. 2019	Not CML treated with TKI and renal outcomes
71	EMBASE	Full-text	Turkina A.G., et al. 2014	Not CML treated with TKI and renal outcomes
